# An efficient tomato-detection method based on improved YOLOv4-tiny model in complex environment

**DOI:** 10.3389/fpls.2023.1150958

**Published:** 2023-04-03

**Authors:** Philippe Lyonel Touko Mbouembe, Guoxu Liu, Jordane Sikati, Suk Chan Kim, Jae Ho Kim

**Affiliations:** ^1^ Department of Electronics Engineering, Pusan National University, Busan, Republic of Korea; ^2^ School of Computer Engineering, Weifang University, Weifang, China

**Keywords:** YOLOv4-tiny model, tomato detection, deep learning, computer vision, agriculture

## Abstract

Automatic and accurate detection of fruit in greenhouse is challenging due to complicated environment conditions. Leaves or branches occlusion, illumination variation, overlap and cluster between fruits make the fruit detection accuracy to decrease. To address this issue, an accurate and robust fruit-detection algorithm was proposed for tomato detection based on an improved YOLOv4-tiny model. First, an improved backbone network was used to enhance feature extraction and reduce overall computational complexity. To obtain the improved backbone network, the BottleneckCSP modules of the original YOLOv4-tiny backbone were replaced by a Bottleneck module and a reduced version of BottleneckCSP module. Then, a tiny version of CSP-Spatial Pyramid Pooling (CSP-SPP) was attached to the new backbone network to improve the receptive field. Finally, a Content Aware Reassembly of Features (CARAFE) module was used in the neck instead of the traditional up-sampling operator to obtain a better feature map with high resolution. These modifications improved the original YOLOv4-tiny and helped the new model to be more efficient and accurate. The experimental results showed that the precision, recall, $F_{1}$ score, and the mean average precision (mAP) with Intersection over Union (IoU) of 0.5 to 0.95 were 96.3%, 95%, 95.6%, and 82.8% for the improved YOLOv4-tiny model, respectively. The detection time was 1.9 ms per image. The overall detection performance of the improved YOLOv4-tiny was better than that of state-of-the-art detection methods and met the requirements of tomato detection in real time.

## 1 Introduction

Recent advances of artificial intelligence technology have allowed wide applications in every area of life, including agriculture. For a decade, fruit detection has been a very active research direction. Detecting and sorting single crops plants such as oranges, apples, tomatoes, etc. are difficult and time intensive due to the number of varieties of the same fruit and environment conditions such as cluster. With the development of artificial intelligence, this can be done by robots. Moreover, Computer vision and related algorithms have been applied to improve the efficiency, intelligence, and remote interactions of robots in complex agricultural environments (Cheng et al., 2020).

A series of traditional fruit detection and recognition algorithms have been proposed. Most of them used non-pattern methods such as color, texture, and geometry methods for fruit detection. Linker et al. (2012) used color and texture to classify green apples. However, sunlight and color-saturation variation which constitute the illumination variation had a large impact on their results. Furthermore, Zhao et al. (2016) developed a method based on segmented mature tomatoes from background using an optimal threshold on fusion image features, but illumination also affected their results. Moreover, an optimal threshold extracted from the intensity histogram of a red-color-difference enhanced image for apple recognition. But this method was restricted to ripe apples which present different color to the background. Liu et al. (2019) proposed a coarse-to-fine method for ripe tomato detection in greenhouse. A naive Bayes classifier combined with a histogram of oriented gradients was applied to recognize tomatoes. The method also used a colour analysis to remove false detection. But due to the low-level abstraction capabilities of hand-crafted features, it was difficult to adapt this method on complex environment changes. Finally, a shape analysis method for mature apple localization proposed by Kelman and Linker (2014) used a canny filter to find the edges in the image. The method also used a pre-processing operation and convexity test to detect the edges that correspond to three-dimensional convex objects. The performance was influenced by illumination and leaves that have similar convex surfaces to apples.

Since the traditional methods were based on handcrafted features, they had several drawbacks, such as low-level of feature extraction in certain conditions. These problems were conquered with the introduction of deep learning (Krizhevsky et al., 2012; Simonyan and Zisserman, 2014). Deep learning techniques have great performance in many fields, including vision tasks (Kamilaris and Prenafeta-Boldu, 2018). First, Sa et al. (2016) merged multi-modal color (RGB) and near-infrared (NIR) information based on a Faster R-CNN (Ren et al., 2015) detector for fruit detection. The model obtained better result than previous models. However, it was difficult to detect small fruits, and the speed still needed to be improved for real-time detection for a harvesting robot. Rahnemoofar and Sheppard (2017) proposed a modified Inception-Resnet architecture (Szegedy et al., 2017) for fruit counting and achieved good results. However, the model just counted fruit and did not detect them. Furthermore, Mu et al. (2020) and Gao et al. (2020) proposed an R-CNN algorithm (Girshick et al., 2014) using ResNet (Szegedy et al., 2017) as a backbone network for the detection, counting and size estimation of green tomatoes. Afonso et al. (2020) used Mask R-CNN (Kaiming et al., 2017) to tomato datasets for detection. Many neural networks were used as backbone to extract feature map.

“You Only Look Once” (YOLO) models were proposed by Redmon et al. (2016); Redmon and Farhadi (2017); Redmon and Farhadi (2018); Bochkovskiy et al. (2020), and Wang et al. (2022) for object detection. They had great improvement in both speed and accuracy compared with the previous region proposal-based detectors (Girshick et al., 2014; Ren et al., 2015; Kaiming et al., 2017), which performed detection in a two-stage pipeline. YOLO models directly predicted the bounding boxes and their corresponding classes with single feed-forward network. There are some studies on fruit detection using YOLO models. Liu et al. (2020) developed a robust model on tomato detection named YOLO-Tomato based on YOLOv3. The traditional rectangular box was replaced with a circular bounding box to match the tomato target. The model achieved an AP of 96.40% with a detection time of 54 ms. Moreover, Fu et al. (2020) developed an algorithm based on improved YOLOv3-tiny to detect kiwifruits in orchard. Xu et al. (2020) proposed a fast method of detecting tomatoes in a complex scene for picking robots, and their experimental results showed that the F1 score was 91.92% with an inferential of 40.35 ms. Furthermore, Wang X et al. (2021) also proposed an algorithm based on YOLOv3-tiny to detect diseases of occlusion and overlapping tomato leaves. A YOLOv3-tiny-IRB algorithm was used to reduce layer-by-layer loss if information during network transmission. The model got a mAP of 93.1%. Furthermore, Arunabha and Jayabrata (2021) proposed a detection method for fine grain based on a modification of YOLOv4 model and achieve good results. In their model, the Dense Net (Gao et al., 2018) architecture was inserted in the backbone to enhance feature map extraction. The result showed the mAP was 96.29% with a detection time of 70.19 FPS. Rupareliya et al. (2022) proposed a deep learning-based tomato detection, where different versions of the YOLO architectures were used. Chenglin et al. (2022) proposed YOLO-PEFL model to detect pear flowers in the natural environment based on improved YOLOv4. The AP of the model was 96.71%, the model size was reduced by 80% approximatively, and the detection time was 0.027 s. Finally, Tang et al. (2023) proposed YOLO- Oleifera based on improved YOLOv4-tiny model and binocular stereo vision, and it achieved an AP of 92.07% with an average of 31 ms to detect each fruit image.

Although much research has been conducted on fruit detection in complex environment, the detection accuracy and the efficiency still need to be improved to meet the requirement of fruit detection under complicated conditions.

**Figure 1:**
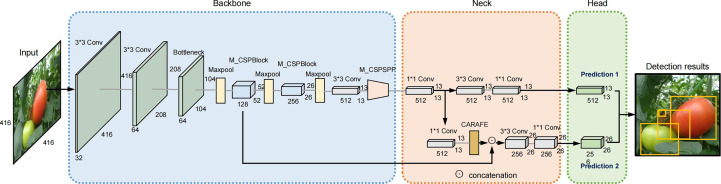
An overview of the improved YOLOv4-tiny model.

To address the above issues, an efficient tomato-detection method was proposed based on an improved YOLOv4-tiny model in this study. **Figure 1** shows an overview of the improved model. The main contributions of this study are as follow:

1 A modified BottleneckCSP module was designed and inserted in the backbone network to enhance the feature extraction and to reduce the computational complexity,
2 A tiny version of CSP-SPP module was also designed and attached to the new backbone network to improve the receptive field,
3 The CARAFE module was used in the neck to get feature map with higher feature map,
4 Extensive experiments were conducted on the tomato datasets to show that the improved YOLOv4-tiny model outperformed the original YOLOv4-tiny model and other state-of the-art object detectors in terms of accuracy (mAP (0.5)) and reached a real-time detection speed.

## 2 Theoretical background

### 2.1 YOLO series

YOLO is a state-of-the-art in real time object detection methods. YOLOv1, YOLOv2, and YOLOv3 (Redmon et al., 2016; Redmon and Farhadi, 2017; Redmon and Farhadi, 2018) were the first versions, and YOLOv2 was proposed with the objective of increasing the accuracy significantly. The idea of anchors for detection introduced in YOLOv2 was inspired by Faster R-CNN. It was also based on some other concepts such as Batch Normalization (Ioffe and Szegedy, 2015) and Skip connection (He et al., 2016). YOLOv3 evolved from YOLOv1 and YOLOv2 and became one of the state-of-the-art methods for object detection. It used Darknet-53 (Redmon and Farhadi, 2018) as a backbone instead of Darknet-19 (Redmon and Farhadi, 2017), multi-scale feature extractors (FPN) (Tsung-Yi et al., 2017), and binary cross-entropy loss instead of Softmax classification loss. YOLOv4 (Boschkovskiy et al., 2020) was released with the aim of improving YOLOv3.

Unlike Faster R-CNN, YOLO uses a different approach by applying a single neural network to a full image. This network divides the input into an S×S grid and performs detection in each cell. Each cell predicts bounding boxes along with the confidence of those boxes. These confidence scores reflect how confident the model is about whether the box contains an object or not. If it is confident, the confidence score tells how accurate the IoU of the ground truth ( GT ) and the predictions ( pred ) is. Equation (1) gives the formula of confidence:


(1) Confidence= P(Object)×IoU(GT, pred)

Where P(Object) ∊ [0,1].

In YOLO model detection, each grid cell predicts C class probabilities for the object, so (5+ C ) values are predicted by each cell: x, y, h, w, Confidence, and C  class probabilities. x and y are the center coordinates of the box, and w and h are the width and the height of the box, respectively.

### 2.2 YOLOv4-tiny architecture

YOLOv4-tiny is a lightweight version of YOLOv4 that makes the network structure simpler and reduces parameters. It can achieve real-time detection. It uses a CSPDarknet-19 (Boschkovskiy et al., 2020) network as a backbone network instead of CSPDarknet-53, which is used in YOLOv4. By removing the computational bottlenecks that have a higher amount of calculation in the CSP-block module, it reduces the amount of calculation while increasing the accuracy. YOLOv4-tiny uses the LeakyReLU function as an activation function to simplify the computation process. Batch Normalization (BN) and Maxpooling are used between the layers of the CNN to speed-up training and select the maximum pixel values of features, respectively.

In the neck, a Feature Pyramid Network (FPN) is used. It can integrate different scales for implementing rich semantic information of a deep network and geometric detail of a shallow network to strengthen the ability of features extractions and to increase the object detection speed. The YOLO head uses features obtained by the FPN to make the final prediction and to form two prediction scales of 13×13 and 26×26 .

### 2.3 Content-aware reassembly of features (CARAFE)

CARAFE (Jiaqi et al., 2019) is a feature map up-sampling operator that has two modules: a kernel prediction module and content-aware module. The kernel prediction module is responsible for generating the reassembly kernel in a content-aware manner. Each source location in the input corresponds to the target location $\sigma^{2}$ in the output. Each target location requires a $k_{up}\times k_{up}$ reassembly kernel, where $k_{up}$ is the reassembly kernel size. It will output the reassembly kernels of size $C_{up}\times H\times W$ , where $C_{up}=\sigma^{2}\times k_{up}^{2}$ .

The kernel prediction module has three sub-modules:

- A channel compressor sub-module reduces the channel of the input feature map by using a convolution layer (from $C\text{ to}\;C_{m})$ with kernel size of 1×1 .

- A content encoder sub-module takes the compressed feature map as input and encodes it to generate reassembly kernels by using a convolution layer of size $k_{encoder}$ . The parameters of the encoder are $k_{encoder}\times k_{encoder}\times C_{m}\times C_{up}$ .

- A kernel normalizer sub-module uses a Softmax function on each reassembly kernel.

The content-aware reassembly module reassembles the features within a local region *via* the function ∅ . This function is just a weighted sum operator. For a target location l′ and a corresponding square region $N\left(Input_{l},k_{up}\right)$ centered at l=(i,j) , the reassembly is shown in Equation (2):


(2) $$Output_{l^{\prime }}=\sum_{n=-r}^{r}\sum_{m=-r}^{r}W_{{l^{\prime }}_{\left(n,m\right)}}\cdot input_{\left(i+n,j+m\right)}$$

where $=\frac{k_{up}}{2}$ , *W*_l′_ is the location-wise kernel for each location l *‘* based on the input, and l *‘* is the neighbor location of l . The semantics of the reassembly feature maps is stronger with CARAFE than the original up-sampling operator because the information from relevant points in a local region is attended. CARAFE has several advantages: a large field of view, a content-aware handling, and it is lightweight and fast to compute.

## 3 Materials and methods

### 3.1 Image acquisition

**Figure 2:**
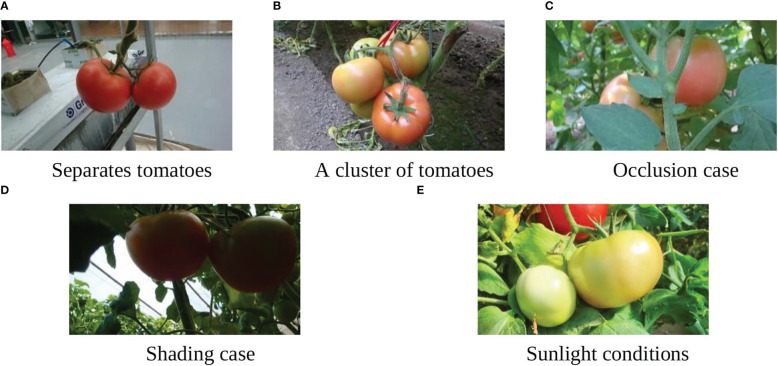
Tomato samples with different growing circumstances. **(A)** Separated tomatoes, **(B)** Cluster of tomatoes, **(C)** Occlusion case, **(D)** Shading case, and **(E)** Sunlight conditions.

The tomato datasets (Liu et al., 2020) used in this research were taken from December 2017 to November 2019 in Vegetable High-Tech Demonstration Park, Shouguang, China (36°51’44.2’’N and 118°49’27.3’’E). The images were taken using a digital camera (Sony DSC-W170, Tokyo, Japan) with a resolution of 3648 × 2056 pixels. The camera has a precision 5× wide-angle zoom Carl Zeiss Vario-Tessar lens with a range equivalent to a 28-140mm zoom on a 35mm camera, which allows it to take shots in tight spaces or get an entire group of things in the frame. Moreover, it incorporates Sony’s Super Steady-shot optical image stabilization to minimize blur caused by camera shake at slow shutter speeds. All the images were taken in natural daylight with different conditions including illumination variation, occlusion, and overlap. A total of 966 images were taken and divided into a training set and a test set. The training set had 725 images and contained 2553 tomatoes, while the test set had 241 images and contained 912 tomatoes. **Figure 2** shows some examples from the datasets under different conditions.

### 3.2 Image augmentation

**Figure 3:**
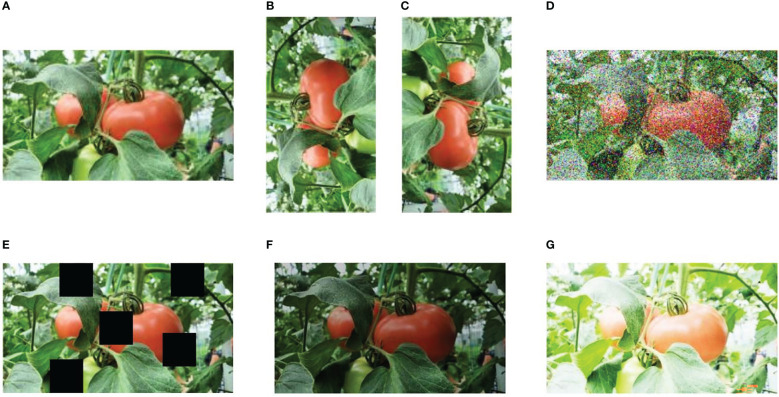
Some examples of image augmentation operations. **(A)** original image, **(B, C)** rotation ( $90^{{}^{\circ}}$ and $270^{{}^{\circ}}$ ), **(D)** noise (salt and pepper), **(E)** cutout with five counts, and **(F, G)** exposure (brightness changes).

To prevent non-convergence phenomenon or over fitting during the training process, in this study, the images were augmented using various data-augmentation methods, such as rotation, noise, brightness transformation, and cutout, as shown in **Figure 3** (Huang et al., 2020; Wu et al., 2020). To help the model to be insensitive to camera orientation, the original images were rotated by $90^{{}^{\circ}}$ and $270^{{}^{\circ}}$ . For the noise, we generated “salt and pepper” noise on the images, which can help the model to be more robust to noise. For the brightness transformation, we randomly changed the intensity of the pixels from -70% to 70%. Finally, a cutout method was adopted to help the model to be more resilient to object occlusion. All these methods were used before training to expand the datasets, which can help the model to be more accurate.

### 3.3 The improved YOLOv4-tiny model architecture

**Figure 4:**
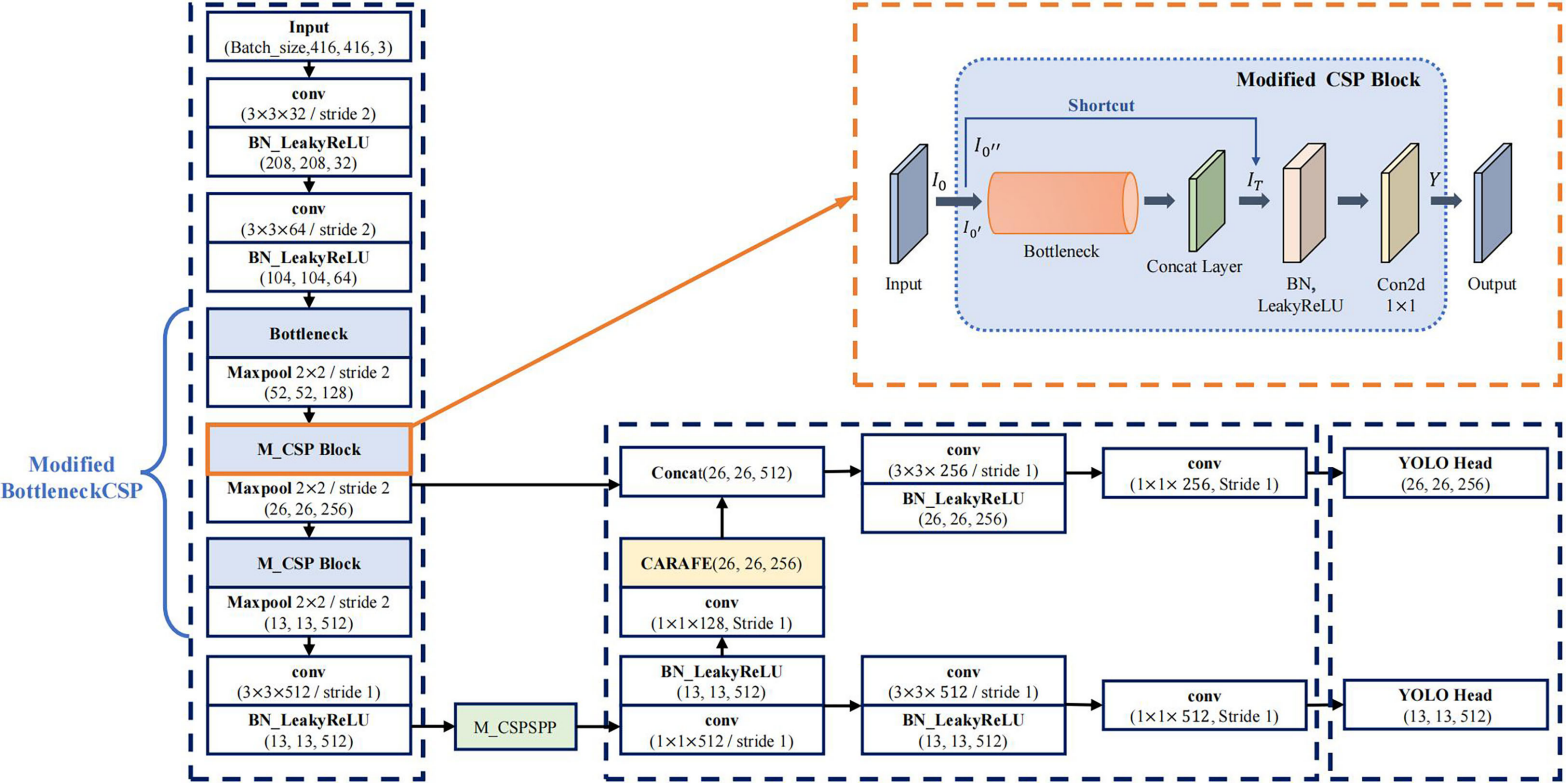
The improved YOLOv4-tiny model architecture.

One of the advantages of YOLOv4-tiny is the fast detection speed because of its simplicity. However, due to the reduction of the number of layers, the feature capability is insufficient, and the feature utilization of the algorithm is low. This leads to low detection accuracy. To solve this issue, we propose a new model based on YOLOv4-tiny. The architecture of the improved model is shown in **Figure 4**.

#### 3.3.1 The modified backbone network

To further improve the detection accuracy and robustness of YOLOv4-tiny model under complex conditions, it is needed to improve the detection accuracy further. The backbone of YOLOv4-tiny contains three BottleneckCSP modules, which consist of multiple convolutional layers, as shown in section 2.2. Even though the convolution operation can extract the features in the image, the convolutional kernel has a large number of parameters, which increases the computation load.

To reduce the number of parameters, the first BottleneckCSP of the original network is replaced with a Bottleneck module (He et al., 2016). Moreover, the original Bottleneck CSP module is modified to enhance feature extraction, capture more information, and reduce the computational complex. The modified BottleneckCSP is simpler, faster, lighter, and has better fusion characteristics.

**Figure 5:**
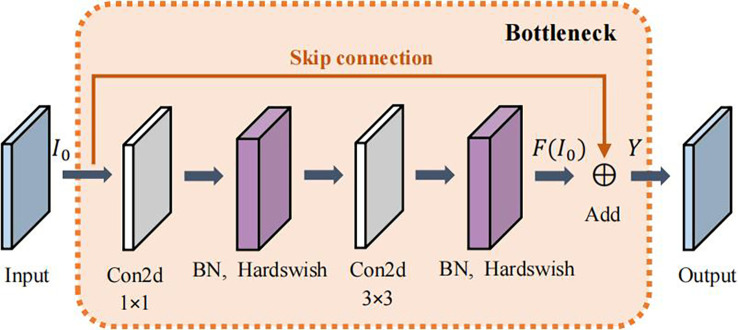
Bottleneck module architecture.

**Figure 6:**
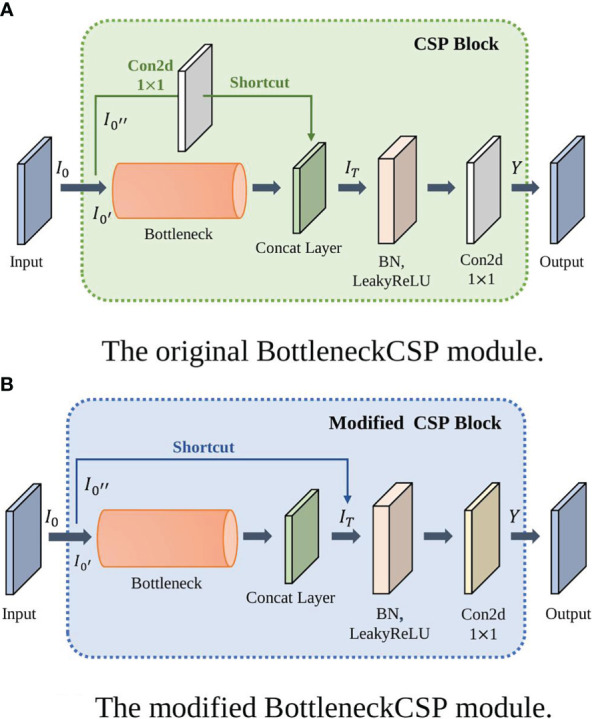
The BottleneckCSP module architectures. **(A)** Original BottleneckCSP, **(B)** The modified module.

The convolutional layer on the bridge branch of the original module was removed so that part of the input of the BottleneckCSP is directly connected to the output feature map of the other branch. This effectively reduces the number of parameters in the module. **Figures 5**, **6** show the bottleneck architecture and the difference between the original BottleneckCSP and the modified one, respectively.

From the original architecture of BottleneckCSP shown in **Figure 6A**, Equations (3) – (5) can be derived:


(3) $$I_{0}=\left\{I_{0'},\;I_{0''}\right\}$$

where $I_{0}$ is the input data, $I_{0'}$ is the first half of the input data, and $I_{0''}$ is the second half of the input data.


(4) $$I_{T}=\left[Bottleneck\left(I_{0'}\right),Conv2d_{1\times 1}\left(I_{0''}\right)\right]$$

where $I_{T}$ is the concatenate layer of $I_{0'}$ and Bottleneck of $I_{0''}$ , and $Conv2d_{1\times 1}$ is a convolutional layer with 1×1 kernel size.


(5) $$Y=Conv2d_{1\times 1}(LeakyReLu,BN\left(I_{T}\right)$$

where Y is the output layer.

Similar to Equation (4), the concatenate layer of $I_{T}$ in the new BottleneckCSP module is represented in Equation (6).


(6) $$I_{T}=\left[Bottleneck\left(I_{0'}\right),I_{0''}\right]$$

The remaining two original BottleneckCSPs are replaced with the modified one in the backbone network to make it more efficient and enhance feature extraction.

**Figure 7:**
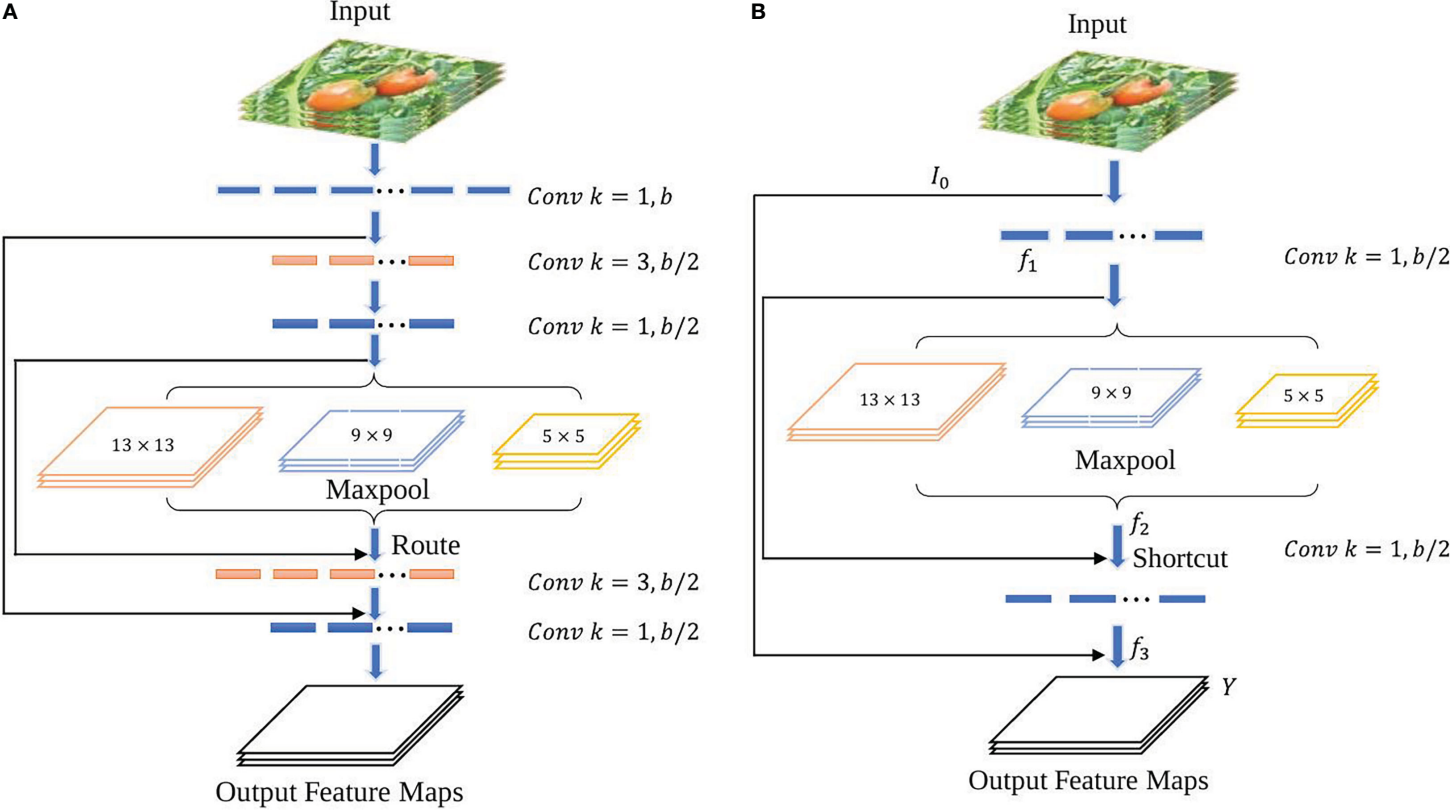
CSP-SPP module architectures. **(A)** the original module used in Scale-YOLOv4, **(B)** The tiny version of the CSP-SPP module.

Scales-YOLOv4 (Wang CY et al., 2021) introduced a CSP-Spatial Pyramid Pooling module (CSP-SPP), which used a cross stage process for down-sampling convolution operations. However, it was designed for large-scale object detection models with large numbers of parameters and is not suitable for a tiny object detection. To adapt it to a tiny object detection, a tiny version of the CSP-Spatial Pyramid Pooling module is proposed in this study. It removes 1×1 and 3×3 convolutional layers to reduce the parameters and increase the accuracy of the model. **Figure 7** shows the architecture of the original CSP-SPP and the tiny version of the module.

Equations (7) – (10) can be derived from the new module shown in **Figure 7B**:


(7) f1=Conv1×1(I02)

(8) $$f_{2}=Maxpooling\left(f_{1}\right)$$

(9) $$f_{3}=Conv_{1\times 1}\left(\left[f_{1},\;f_{2}\right]\right)$$

(10) $$Y=Conv_{1\times 1}(\left[I_{0},\;f_{3}\right]$$

Where $Conv_{1\times 1}$ is a convolutional layer with 1×1 kernel size, $f_{1},\;f_{2},$ and $f_{3}\;$ are feature maps, and Y is the output layer.

#### 3.3.2 The modified neck network

**Figure 8:**
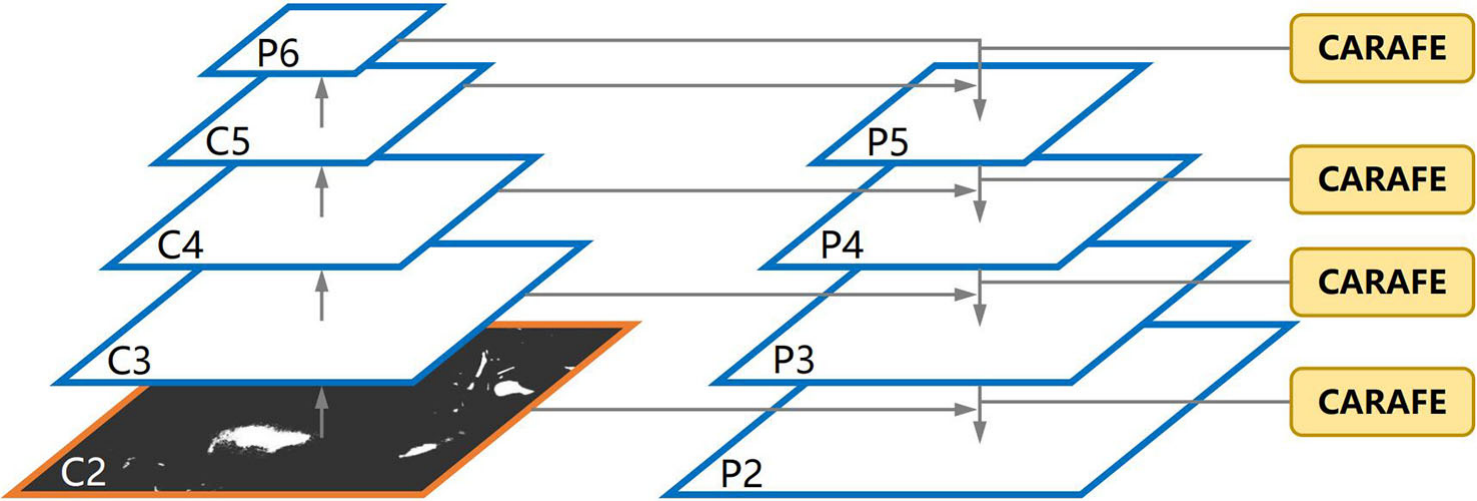
The FPN architecture with CARAFE.

In the YOLOv4-tiny neck, FPN (Tsung-Yi et al., 2017) is used to construct a feature pyramid of strong semantics with a top-down pathway and lateral connections. In the top-down pathway, a low-resolution feature map is firstly up-sampled twice with the nearest neighbour interpolation and then fused with a high-resolution one. It adopts spatial distance between pixels to guide the up-sampling process, but it considers only sub-pixel neighbours and fails to capture the rich semantic information required by dense prediction tasks. In Pixel shuffle (Shi et al., 2016) up sampling method, the feature map is extracted using sub-pixel convolution and then expands by a dimensional space. However, it scales the image size without changing the current amount of feature information. To solve this issue, all feature levels is substituted with CARAFE (Jiaqi et al., 2019), as shown in section 2.3. **Figure 8** shows the new architecture. CARAFE is a region content-based up sampling method that first gets the up-sampling kernel in the up-sampling kernel prediction module, and uses it to up sample the corresponding positions of the original map. Then the new feature is used in the feature reassembly module to complete the up-sampling process and gets better output feature with high resolution. In addition, the kernel prediction module normalizes the features in the up-sampled region to maintain a constant value after up sampling, thereby reducing distortion. This modification is smooth, and no extra change is required. Moreover, it occupies less computing power, is lighter, and has demonstrated good performance in object detection and semantic segmentation tasks.

### 3.4 Experimental setup

In this study, the computer used had Intel i5, 64-bit 3.30-GHz quad-core CPUs (Santa Clara, CA USA), 16 GB of RAM, and an NVIDIA GeForce GTX 1070Ti GPU. The model framework was Pytorch with related software CUDA 11.1 and Python 3.8.10. The batch size was set to 8. The input image size was: 416×416 . The setting of some hyper parameters used in this study is given as follows: number of epochs: 400, learning rate: 0.001, optimizer weight decay: 94.75, STD momentum: 96.3, warm-up initial momentum: 0.8, batch size: 8, box loss gain: 0.05, classification loss gain: 0.5, cls BCE loss positive weight: 1.0, object loss gain: 1.0, and anchor multiple threshold: 4.0.

#### 3.4.1 Evaluation metrics

Evaluation indicators (Padilla et al., 2020) such as precision, recall, mAP, and $F_{1}$ score were used to evaluate the model performance. The indicators are defined as follows:


(11) $$Recall=\frac{TP}{TP+FN}$$

(12) $$Precision=\frac{TP}{TP+FP}$$

where TP, FN,  and FP are abbreviations for true positive (correct detection), false negative (miss), and false positive (false detection), respectively. The mAP was adopted to show the overall performance of a model under different confidence thresholds. It is defined as follows:


(13) $$mAP=\frac{1}{N_{classes}}\sum_{a=1}^{N_{cls}}AP_{a}$$

with


(13.a) $$AP=\sum_{q}^{Q}(r_{q+1}-r_{q})\underset{\tilde{r}\geq r_{q+1}}{max}p(\tilde{r})$$

where $p\left(\tilde{r}\right)$ is the measured precision at recall $\tilde{r}$ , and $N_{cls}$ is the number of classes. The $F_{1}$ score is defined as follows:


(14) $$F_{1}=\frac{2\times Recall\times Precision}{Recall+Precision}$$

#### 3.4.2 Loss function

The loss function in this study considered the regression error of bounding coordinates, the confidence error of bounding box, and the classification error of object category. Equation (15) below shows how we calculated the loss function:


(15) $$Loss=Loss_{reg}+Loss_{conf}+Loss_{cls}$$

- Loss regression:


(15.a) $$Loss_{reg}=1-IoU+\frac{d^{2}\;\left(\hat{b},b_{gt}\right)}{c^{2}}+\alpha v$$

with


(15.b) $$IoU=\;\frac{\hat{b}\cap^{}b_{gt}}{\hat{b}\cup^{}b_{gt}}$$

and


$$v=\frac{4}{\pi^{2}}{\left(tan^{-1}\frac{w_{gt}}{h_{gt}}-tan^{-1}\frac{w}{h}\right)}^{2},$$

(15.c) $$\alpha =\frac{v}{\left(1-IoU\right)+v}$$

where b and $b_{gt}$ are predicted bounding boxes and ground truth bounding boxes, respectively, d is the distance between the predicted center point and the true center point, c is the diagonal length of the enclosing box covering b and $b_{gt}$ , and α and v are the positive trade-off and aspect ratio parameter, respectively.

From the equations above, we can see that the loss regression function works from three aspects: the overlap area, centroid distance, and the aspect ratio between the bounding box and the ground truth.

- Loss confidence:

To know the confidence loss, we need to calculate the confidence of the grid cell.


(15.d) $$C=P\left(object\right)\times IoU\left(b,b_{gt}\right)$$

then,


$$Loss_{conf}=\sum_{i=1}^{s\times s}\sum_{j=1}^{NB}\lambda_{i,j}[C_{i}{\;}_{\dot{n}}log({\tilde{C}}_{i})log\left(1-C_{i}\right)]$$

(15.e) $$-\sum_{i=i}^{s\times s}\sum_{j=1}^{NB}(1-\lambda_{i,j})\left[C_{i}\cdot \log{\tilde{C}}_{i}+(1-C_{i})\log(1-{\tilde{C}}_{i}\right]$$

with


(15.f) $$\lambda_{i,j}=\{\begin{array}{c} 1,\;if\;part\;of\;j-th\;bounding\;box\;is\;in\;the\;i-th\;grid\;cell\\ 0,\;otherwise \end{array}$$

where s×s is the grid cell size, NB is the number of bounding boxes, ${\tilde{C}}_{i}$ is the obtained confidence from prediction box, and $C_{i}$ is the confidence threshold.

- Loss classification:


(15.g) $$Loss_{cls}=\sum_{i=1}^{s\times s}\sum_{j=1}^{NB}\lambda_{i,j}\sum_{a\in classes}\left[p_{i}(a)\log({\tilde{p}}_{i}(a))+(1-p_{i}(a))\log(1-\tilde{p}(a))\right]$$

where $p_{i}$ is the true probability of detecting the object, ${\tilde{p}}_{i}$ is the probability score from the prediction, and a is a class associated with target detection. The loss function of YOLOv4-tiny converged gradually in the training process, such that the position and confidence of the bounding box are close to the ground truth.

## 4 Results and discussions

### 4.1 Ablation study

In this study, three major modifications were studied before obtaining the final result. **Table 1** shows the ablation analysis of the different modifications. An ablation analysis of the impact of different modifications to the original YOLOv4-tiny was performed. **Table 1** shows exactly what modifications were made.

**Table 1:** Ablation analysis of the different modifications.

	Modified BottleneckCSP	Modified CSP-SPP	CARAFE	mAP (0.5:0.95) (%)	Time (ms)
Modification				78.4	3.5
	✔			80.1	2.6
	✔	✔		82.0	1.9
	✔		✔	81.6	2.3
	✔	✔	✔	82.8	1.9

First, the modified BottleneckCSP was incorporated into the backbone instead of the original BottleneckCSP module, which increased the accuracy by 1.7% and reduced the time by 0.9 ms compared to the original YOLOv4-tiny. Second, the tiny CSP-SPP module was attached to the modified backbone, which contributed another 1.9% improvement to the accuracy, and the time was reduced by 0.7 ms. Lastly, when the CARAFE module was adopted in the neck, the accuracy was further increased by 0.8%.

**Figure 9:**
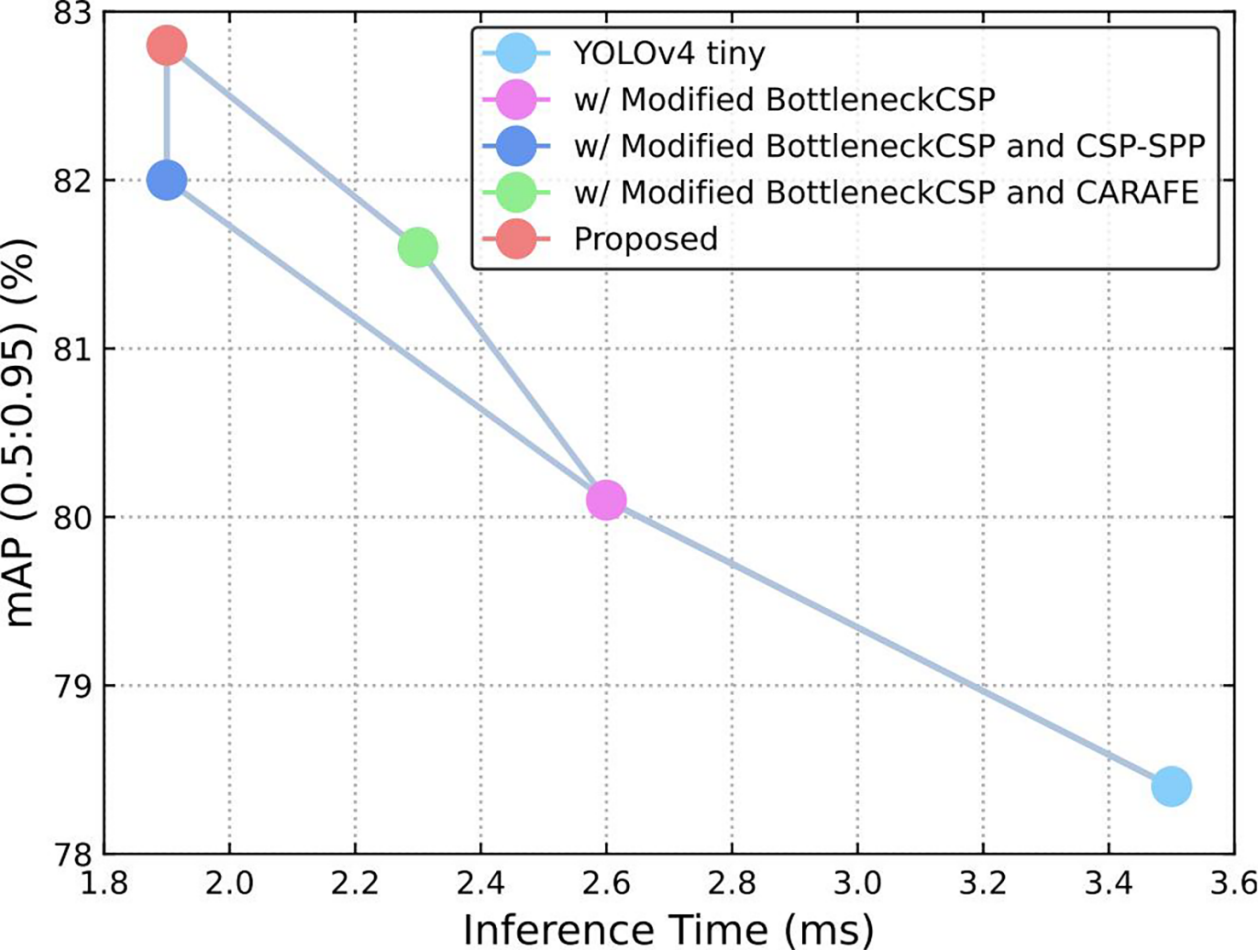
Speed (ms) versus accuracy (mAP).

Also, we tested the function of the CARAFE module based on the modified BottleneckCSP and found that it improved the accuracy by 1.5%, which is a little lower than that of the CSP-SPP module. This showed the efficiency and effectiveness of each modification. In accordance with **Table 1**, **Figure 9** shows that the accuracy and speed were both improved with the proposed modification.

Moreover, experiment was also performed with Pixel shuffle up sampling method (Shi et al., 2016). It implements sub-pixel method convolution to extract the feature map and then expands it by a dimensional space to obtain the up-sampling results. Compared with CARAFE, the Pixel shuffle has an accuracy of 81.03% and a detection time of 2.2ms, which are both worse than that of CARAFE.

### 4.2 Feature map visualization

**Figure 10:**
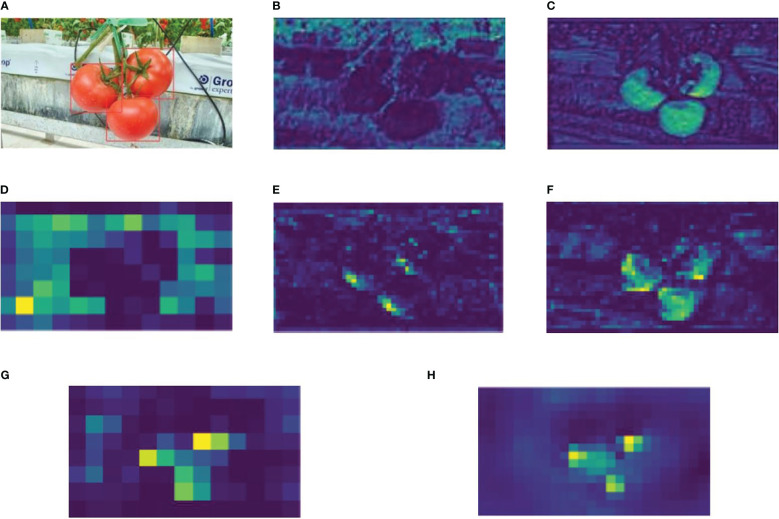
**(A)** The labeled input image, **(B)** 29^th^ feature of the first BottleneckCSP of YOLOv4-tiny, **(C)** 29^th^ feature of the Bottleneck module of the improved method, **(D)** 2^nd^ feature of the modified CSP-SPP module, **(E)** 2^nd^ feature of the second BottleneckCSP, **(F)** 2^nd^ feature of the modified BottleneckCSP, **(G)** 29^th^ feature of original up-sampling operator, **(H)** 29^th^ feature of CARAFE operator.

Features were visualized in some stages of the algorithms (original YOLOv4-tiny and the improved YOLOv4-tiny). **Figure 10** focuses on features where the original model was modified. **Figure 10A** shows an input image with tomatoes labeled for better visualization. **Figures 10B, C** represent stage 2 of both the original algorithm (the first BottleneckCSP module) and the modified algorithm (Bottleneck module), respectively. **Figure 10D** shows the second feature of the modified CSP-SPP module.

Stage 4 is shown in **Figures 10E, F** and represents the second BottleneckCSP module of the original algorithm and the modified Bottleneck module. Finally, **Figures 10G, H** show features in the original up-sampling operator in the neck and the CARAFE operator module, respectively. Moreover, CARAFE has a large field of view and can effectively aggregate context information, resulting in a good feature map. It can be seen in **Figure 10H**. Combining all the visualization in **Figure 10**, each modification has better features with high resolution than features in the original algorithm, which means that the improved model is better and more efficient than the original model.

### 4.3 Comparison of the improved YOLOv4-tiny with different one-stage detection algorithms

The performance of the improved YOLOv4-tiny was compared with other one stage detection algorithms: MobileNetv1 (Andrew et al., 2017), YOLOv3-tiny (Redmon and Farhadi, 2018), ShuffleNetv2 (Ma et al., 2018), MobileNetv3 (Howard et al., 2019), and YOlOv4-tiny (Boschkovskiy et al., 2020). **Table 2** shows that the improved YOLOv4-tiny model has the best detection performance among all the methods. The mAP (0.5:0.95) was 7.4%, 11.5%, 6.2%, 5.4%, 0.8% and 4.4%, higher than those of Mobilenetv1, YOLOv3-tiny model, ShuffleNetv2, MobileNetv3, YOLOv5s, and YOLOv4-tiny model, respectively. The average detection time of the improved method was 1.9 ms, which met the requirement of real-time fruit detection.

**Table 2:** A comparison of the different models.

Model	Precision (%)	Recall (%)	F1 (%)	mAP (0.5) (%)	mAP (0.5:0.95) (%)	GFLOPs	Time (ms)
MobileNetv1*	95.1	91.1	93.0	96.5	75.4	3	1.7
YOLOv3-tiny	95.1	91.9	93.4	97.4	71.3	10	3.8
ShuffleNetv2*	94.1	92.8	93.4	96.6	76.6	7	1.7
MobileNetv3*	96.4	90.9	93.5	96.8	77.4	8	1.6
YOLOv5s	96.3	94.2	95.2	98.3	81.7	16.8	2.7
YOLOv4-tiny	95.3	94.0	94.6	98.0	78.4	6.8	3.5
The Improved Yolov4-tiny	96.3	95.0	95.6	98.5	82.8	9	1.9

*MobileNetv1, Shufflenetv2, and MobileNetv3 were used as backbone network and YOLOv4-tiny head was used for detection.

As shown in **Table 2**, compared with MobileNetv1, YOLOv3-tiny, ShuffleNetv2, and YOLOv4-tiny, the precision of the improved model increased by 1.2%, 1.2%, 2.2% and 1.0%, respectively. However, the recall increased by 3.9%, 3.1%, 2.2%, and 1.0%, respectively. MobileNetv3 had almost the same precision with the improved model, whereas recall decreased by 4.1%. The F1 score and the mean average precision with IoU of 0.5 increased by 1.0% and 0.5% compared with that of the original YOLOv4-tiny model. Although the detection time of the improved model was slightly lower than that of MobileNetv1 and shuffleNetv2, the improvement of his accuracy was far better than that of MobilenetV1 and ShuffleNetv2. Moreover, the mAP (0.5:0.95) of the improved model is 0.8% higher than the one of YOLOv5s model, with less detection time. Performance of the Improved Model under Different Conditions

To evaluate the performance of the improved YOLOv4-tiny model under different lighting and occlusion environmental conditions, the tomatoes were divided into different groups. According to different lighting conditions, the tomatoes were divided into sunlight and shading groups. Among all the 912 tomatoes, 487 of them are in sunlight conditions and 425 of them are in shading conditions. According to the degree of occlusion or overlap conditions, the tomatoes were divided into slight and severe occlusion cases. Severe cases refer to tomatoes being occluded by leaves, branches or other tomatoes by more than 50% degree.

**Table 3** shows the evaluation results of the improved model under sunlight and shading conditions. 95.1% of the tomatoes were correctly detected under sunlight conditions while 94.8% for shading cases. The missed rates are 4.9% and 5.2% for sunlight and shading cases, respectively. Moreover, the false identification rates are 3.7% and 3.6% for sunlight and shading cases. This means that some leaves, branches or other background are falsely detected as tomatoes, especially when some background presents both similar color and shape as tomatoes.

**Table 3:** Performance of the improved model under different lighting conditions.

Conditions	Tomato Count	Correctly Identified		Falsely Identified		Missed	
		Amount	Rate (%)	Amount	Rate (%)	Amount	Rate (%)
Sunlight	487	463	95.1	18	3.7	24	4.9
Shading	425	403	94.8	15	3.6	22	5.2

Similarly, **Table 4** shows the evaluation results of the improved model under slight and severe occlusion cases. Under slight occlusion case, 95.2% of the tomatoes were correctly detected, and 94.4% were detected under severe occlusion case. The results show that most of the tomatoes could be detected by our improved model except that are severely occluded by other objects. For the false identification rate, the results are 3.2% and 4.7%, respectively.

**Table 4:** Performance of the improved model under different occlusion conditions.

Conditions	Tomato Count	Correctly Identified		Falsely Identified		Missed	
		Amount	Rate (%)	Amount	Rate (%)	Amount	Rate (%)
Slight occlusion	609	580	95.2	19	3.2	29	4.8
Severe occlusion	303	286	94.4	14	4.7	17	5.6

### 4.4 Qualitative analysis of different one-stage models

**Figure 11:**
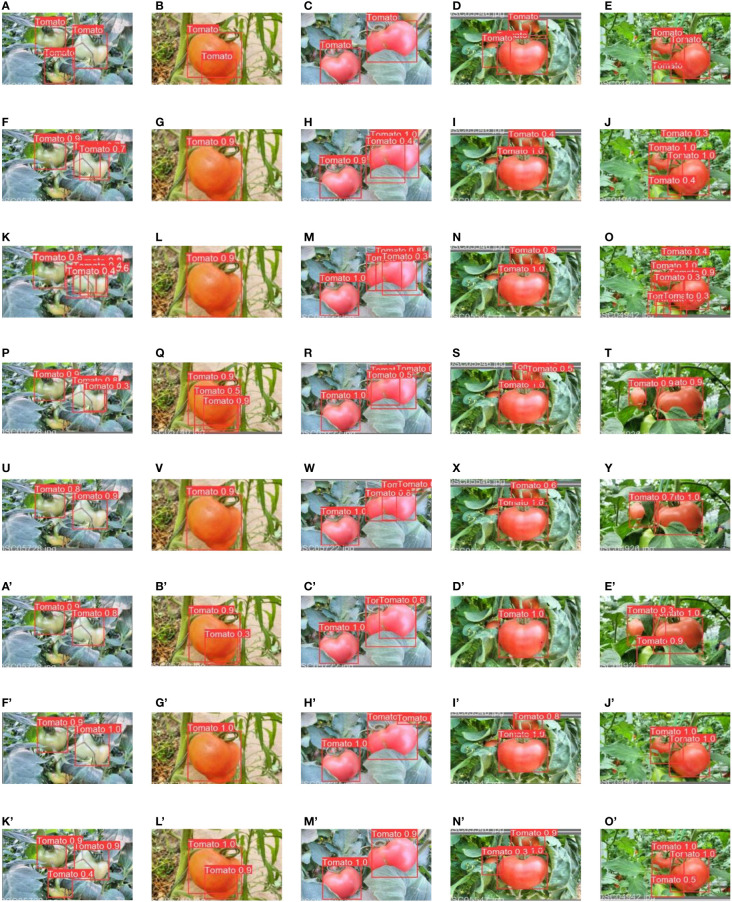
Detection results of different models: **(A–E)** are the labeled images, **(F–J)** are prediction images from MobileNetv1, **(K–O)** are prediction image from YOLOv3-tiny, **(P–T)** are prediction images from ShuffleNetv2 detection, **(U–Y)** are prediction images from MobileNetv3 detection, **(A’–E’)** are the prediction images from YOLOv5s detection, **(F’–J’)** are the prediction images from YOLOv4-tiny detection, **(K’–O’)** are the prediction images from the improved YOLOv4-tiny detection. (* MobileNetv1, ShuffleNetv2, and MobileNetv3 were used as backbone network and YOLOv4-tiny head was used for detection).

**Figure 11** shows the prediction images of the comparison models, respectively. As shown in **Figure 11**, compared to the improved YOLOv4-tiny model, the other detection models have some either missed detections or false detections.

Moreover, the detection performance of the improved YOLOv4-tiny model was better and more efficient than that of the other detection models. The mean average with IoU of 0.5 to 0.95 increased by 4.4% compared to that of the original YOLOv4-tiny model, and the detection time per image was reduced by 1.6 ms. This means that the improved model is more accurate, compact and efficient for fruit detection in complex environment.

### 4.5 Comparison of the improved model with two-stage detection models

The performance of the improved model was compared with that of Faster R-CNN (Ren et al., 2015) and Dynamic R-CNN (Zhang et al., 2020), which are two-stage detection algorithms. **Table 5** shows that Faster R-CNN took much time which led to huge amount of computation, whereas mAP with IoU of 0.5 to 0.95 of Faster R-CNN was 1.1% higher than that of the improved model. The detection time of the improved model was two time less than the detection time of Faster R-CNN. Moreover, the mAP with IoU of 0.5 to 0.95 of the improved model is 4.8% higher than that of the Dynamic R-CNN, with less time of detection. In summary, for the requirement of fruit detection which are accurate detection and a low amount of computation, the improved model is much better than the two-stage detection algorithm.

**Table 5:** A comparison of the improved model and two stage detection model.

Model	Precision (%)	Recall (%)	F1 (%)	mAP (0.5) (%)	mAP (0.5:0.95) (%)	Time (ms)
Faster R-CNN(VGG-16)	96.5	94.8	95.6	97.8	83.9	3.9
Dynamic R-CNN	95.3	93.2	94.2	96.6	78.0	2.4
The Improved YOLOv4-tiny	96.3	95	95.6	98.5	82.8	1.9

## 5 Conclusions and future work

To realize the application of tomato detection under complex environments, it needs a robust and efficient detection algorithm which is both accurate and fast. However, the existing methods are either inaccurate or slow for tomato detection, which cannot satisfy the requirement of tomato detection in the real natural environment. Thus, this study aims at proposing an efficient tomato detection algorithm based on YOLOv4-tiny, to obtain a more robust, fast and accurate tomato detection performance under complex environment conditions. To make the model more efficient, a modified backbone was proposed. The BottleneckCSP modules were replaced in the original backbone with a Bottleneck and modified BottleneckCSP modules to enhance feature extraction and reduce the computational complex. Moreover, a light version of the CSP–SPP module was attached to the modified backbone to improve the receptive field. Finally, to obtain a better feature map with high resolution, the traditional up-sampling operator in the neck was replaced by CARAFE.

Extensive experiments were conducted to verify the performance of the improved model. An ablation study proved the effectiveness of each modification. With the above modifications, the mAP (0.5:0.95) were increased by 1.7%, 1.9% and 0.8%, respectively, showing that the detection performance was greatly improved. The precision, recall, F1 score, mAP (0.5), and mAP (0.5:0.950) were 96.3%, 95.0%, 95.6%, 98.5%, and 82.8%, respectively. The detection speed reached 1.9 ms per image.

Furthermore, the performance of the improved method under different lighting and occlusion conditions were evaluated. The performance of the model was comparable under sunlight and shading conditions, showing that the model was robust to illumination variation. However, the model showed a divergence under different occlusion conditions. Under slight occlusion, 95.2% of the tomatoes were correctly detected, while 94.4% were detected under severe occlusion case. This showed that occluded and overlapped tomatoes could cause inaccurate detections, especially when the occlusion degree exceeds 50%.

The improved YOLOv4-tiny model was compared with some other state-of-the-art algorithms. The results showed that the improved model performed better than the other one-stage models. Moreover, the improved algorithm was compared with two-stage object detection algorithms (Faster R-CNN and Dynamic R-CNN). The results showed that the detection accuracy of the improved model could match that of the two-stage detection models and was faster. This indicates great potential of the improved model for tomato detection in complex environment.

In future work, based on the proposed model in this study, the information about tomato ripeness will be incorporated to classify a tomato in different growing stages. Moreover, further research will be conducted to improve the accuracy for severely occluded tomatoes.

## Data availability statement

The original contributions presented in the study are included in the article/supplementary material. Further inquiries can be directed to the corresponding authors.

## Author contributions

PLTM conceived the idea. PLTM and GL designed the methodology. PLTM and JS performed the experiments and analysis. PLTM wrote the original draft. JK and SK revised the manuscript. JK and GL supervised the experiments. All authors contributed to the article and approved the submitted version.
